# A study on the two binding sites of hexokinase on brain mitochondria

**DOI:** 10.1186/1471-2091-8-20

**Published:** 2007-10-20

**Authors:** Abolfazl Golestani, Hassan Ramshini, Mohsen Nemat-Gorgani

**Affiliations:** 1 Department of Clinical Biochemistry, School of Medicine, Medical Sciences/University of Tehran, Tehran 14155-6447, Iran; 2 Institute of Biochemistry and Biophysics, University of Tehran, Tehran 13145-1384, Iran; 3 Stanford Genome Technology Center, Stanford University, Palo Alto, CA, USA

## Abstract

**Background:**

Type I hexokinase (HK-I) constitutes the predominant form of the enzyme in the brain, a major portion of which is associated with the outer mitochondrial membrane involving two sets of binding sites. In addition to the glucose-6-phosphate (G6P)-sensitive site (Type A), the enzyme is bound on a second set of sites (Type B) which are, while insensitive to G6P, totally releasable by use of high concentrations of chaotropic salts such as KSCN. Results obtained on release of HK-I from these "sites" suggested the possibility for the existence of distinct populations of the bound enzyme, differing in susceptibility to release by G6P.

**Results:**

In the present study, the sensitivity of HK-I toward release by G6P (2 mM) and a low concentration of KSCN (45 mM) was investigated using rat brain, bovine brain and human brain mitochondria. Partial release from the G6P-insensitive site occurred without disruption of the mitochondrial membrane as a whole and as related to HK-I binding to the G6P-sensitive site. While, as expected, the sequential regime release-rebinding-release was observed on site A, no rebinding was detectable on site B, pre-treated with 45 mM KSCN. Also, no binding was detectable on mitochondria upon blocking site A for HK-I binding utilizing dicyclohexylcarbodiimide (DCCD), followed by subsequent treatment with KSCN. These observations while confirmed the previously-published results on the overall properties of the two sites, demonstrated for the first time that the reversible association of the enzyme on mitochondria is uniquely related to the Type A site.

**Conclusion:**

Use of very low concentrations of KSCN at about 10% of the level previously reported to cause total release of HK-I from the G6P- insensitive site, caused partial release from this site in a reproducible manner. In contrast to site A, no rebinding of the enzyme takes place on site B, suggesting that site A is 'the only physiologically-important site in relation to the release-rebinding of the enzyme which occur in response to the energy requirements of the brain. Based on the results presented, a possible physiological role for distribution of the enzyme between the two sites on the mitochondrion is proposed.

## Background

The type I isoenzyme of mammalian hexokinase (HK-I; ATP: D-hexose-6-phoshotransferase, EC 2.7.1.1) binds reversibly to the outer mitochondrial membrane, a process which has been suggested to be involved in regulation of its activity [1]. Accordingly, in a variety of situations, it has been observed, that the rate of glycolysis depends on the level of hexokinase bound to mitochondria [2]. An outer mitochondrial membrane protein responsible for specific binding of HK- I was first isolated by Felgner et al. [3]. Later, this protein was shown to be identical to mitochondrial porin [4] also called voltage-dependent anion channel (VDAC). The protein has been shown to form the channel through which metabolites enter and exit the mitochondrion. It is by this mechanism that the enzyme gains preferential access to mitochondrially-generated ATP, with decreased susceptibility to product inhibition and proteolytic digestion. Thus definition of the molecular basis of the interaction of HK-I and the outer mitochondrial membrane is directly relevant to our understanding of regulation of enzyme activity. A 15-amino acid hydrophobic segment of HK-I is the docking domain necessary and sufficient for binding to mitochondria [5]. Treatment of intact mitochondria with dicyclohexylcarbodiimide (DCCD) was found to produce a large steric hindrance to the interaction between this N-terminal segment and the corresponding region in porin, thereby resulting in inhibition of binding [6]. It has been suggested that changes in HK-I distribution may constitute a target for a new therapeutic approach for malignant tumors [7].

The distribution of the enzyme between mitochondrially bound and dissociated forms has been found to be influenced by the level of certain metabolites, especially G6P [8]. In addition to this, a second type of binding site has been shown to be present in mammalian mitochondria [9], including normal and tumoral human brain tissues [10], which is insensitive to G6P but is released by chaotropic salts such as KSCN [9]. Results obtained on release of HK-I from these "sites" suggested the possibility for existence of distinct populations of the bound enzyme in various species, differing in susceptibility to release by G6P [9].

In the present study, the sensitivity of HK-I toward release by 2 mM G6P (Type A sites) and 45 mM KSCN (Type B sites) has been investigated. Preliminary experiments using rat brain and bovine brain mitochondria indicated that this low concentration of the chaotropic salt is capable of causing partial release of the enzyme from the G6P- insensitive sites (B-HK-I) without disruption of the mitochondrial membrane. Rebinding experiments performed using intact and DCCD-blocked mitochondria, treated sequentially with G6P and KSCN, suggested that the reversible association of the enzyme on mitochondria may be uniquely related to the Type A sites. Use of a low concentration of KSCN appears to have provided a new tool in furnishing information on the properties of the two types of binding sites of HK-I on the mitochondria.

## Results

### Solubilization of mitochondrial hexokinase by G6P and KSCN

Sequential treatment of rat, bovine and human brain mitochondria with 2 mM G6P and 45 mM KSCN was carried out. The extent of release by use of the low concentrations of KSCN was limited but significant and reproducible in all the three cases. Treatment of intact or KSCN- treated bovine or human brain mitochondria with G6P provided similar results, based on units of releasable activity (Table 1). Therefore, it appears that use of 45 mM KSCN does not affect release of the enzyme from Site A of these mitochondria, which occurs by treatment of intact mitochondria with G6P. In rat brain mitochondria, however, presumably due to the presence of large proportion of HK at site A, some of the enzyme at this site is susceptible to release by low concentration of KSCN (Table 1).

**Table 1:** Solubilization of mitochondrially- bound hexokinase of rat, bovine and human brain tissues' mitochondria by sequential treatment with G6P and KSCN

	Treatment	Rat	Bovine	human
% Release	First, with G6P	80 ± 2.1	46 ± 0.8	55 ± 1.8
	Second, with KSCN	10 ± 0.8	9.2 ± 0.75	7.7 ± 1.85
	
	First, with KSCN	33.4 ± 0.8	8.8 ± 0.3	5.1 ± 0.075
	Second, with G6P	51 ± 7.6	44.2 ± 4.3	53 ± 0.95

Solubilization of hexokinase by G6P (2 mM) and KSCN (45 mM) was determined as noted under Methods. The remaining activity of HK on G6P treated mitochondria (*e.g*. for rat; 20%), was subject to release by KSCN. Ten percent of the enzyme activity could be released in this process. Total activity which could be released in the two steps, never exceed 100%. Values shown are means ± SE of at least 3 determinations.

As expected, the extents of release from intact mitochondria, or G6P-treated mitochondria on which HK-I was rebound, were almost identical, when subsequent treatment with G6P was carried out.

### Rebinding of hexokinase on mitochondria

It was expected that treatment by KSCN of mitochondria pre-treated by G6P would enhance the extent of rebinding by providing additional binding sites. However, this was not what was observed and repeated experiments indicated that rebinding on these mitochondria was slightly lower, if any difference were to be suggested from the data (Table 2). The most unexpected results were obtained when rebinding experiments were performed on mitochondria treated with KSCN, prior to treatment with G6P. As indicated (Table 2), no rebinding was detected on these mitochondria. Subsequent treatment of these mitochondria (KSCN-treated) with G6P followed by rebinding experiments indicated somewhat less but similar extents of binding, as compared with the situation in which they were pre-treated with G6P, alone or with G6P followed by KSCN (Table 2). These experiments indicate that site A retains its overall capacity for binding after treatment with 45 mM KSCN and, taken together, suggest a distinct role for the chaotropic salt, at the low concentration utilized, in releasing the enzyme from site B. This somewhat unexpected effect of low KSCN, combined with other experimental finding suggest the proposition that the observed rebinding is related only to the Type A site. Treatment of rat liver mitochondria with up to 60 mM concentration of KSCN provided exactly the same extent of binding (Table 3) further supporting the view that loss of binding (for rat and bovine brain mitochondria, Table 2) was not due to the effect of the chaotropic salt on the mitochondrial membrane.

**Table 2:** Rebinding of rat brain hexokinase to rat and bovine brain mitochondria

	Treatment	Rat	Bovine
% Rebinding	First, with G6P	61 ± 3.9	52 ± 1.4
	Second, with KSCN	57 ± 1.9	48 ± 1.4
	
	First, with KSCN	N.D	N.D
	Second, with G6P	44.5 ± 3.5	36.5 ± 2.1

Rebinding of 0.016 U of rat brain hexokinase to mitochondria previously treated sequentially with 2 mM G6P/45 mM KSCN, and in the reverse order, was carried out. An amount of mitochondrial suspension corresponding to 1 mg protein was used in a total volume of 500 μL in all cases. Values shown are means ± S.E. of at least 3 determinations. Further details are described in Methods section. N.D.; not detectable

**Table 3:** Effect of KSCN at various concentrations on rebinding capacity of rat liver mitochondria

KSCN(mM)	0	35	45	55	65
Rebinding (%)	50.2 ± 2.2	49 ± 1.76	49 ± 0.75	50 ± 2.1	49.5 ± 1.4

Rat liver mitochondria were treated with KSCN at various concentrations. 0.1 unit of hexokinase was rebound on 4.5 mg mitochondria at total volume of 1.00 ml and incubated for 30 minute on ice in the presence of MgCl_2 _(3 mM). The percentage of rebound enzyme was evaluated by the activity rebounded on mitochondria which was assayed according to Methods.

Another approach taken in these rebinding experiments involved treatment of mitochondria with DCCD, which has been previously shown to block site A for HK-I binding [6]. Results presented in Table 4 report on hexokinase binding experiments using initial treatment with G6P followed by blockage with DCCD and, finally, treatment with KSCN. No binding could be detected, thus supporting the proposition that process of release-rebinding of HK-I on mitochondria is uniquely related to site A.

**Table 4:** Use of DCCD in release rebinding experiments involving rat brain HK and rat, bovine and human brain mitochondria

Steps	1	2	3	4
Process	%Release (with G6P)	Blocking (with DCCD)	%Release (with KSCN)	Rebinding
Rat	81 ± 3	Yes	30 ± 2.1	N.D.
Bovine	38 ± 2.7	Yes	19 ± 1.7	N.D.
Human	48.5 ± 3.5	Yes	17.5 ± 2.5	N.D.

Mitochondria from three species were subjected to HK release by G6P (2 mM) in the first step. Then, the exposed binding sites of G6P – treated mitochondria were blocked by DCCD (0.01 mM). At the third step, the remaining HK activity on this DCCD-treated mitochondria was released with 45 mM KSCN. Finally the depleted (both sites) blocked (G6P-sensitive sites) mitochondria were used for the rebinding process, according to details described under Methods. N.D.; not detectable

## Discussion

Over 80% of HK-1 activity in the brain is associated with mitochondria [11]. This interaction has been suggested to play a central role in regulation of HK-I activity [12] and to involve two types of sites [9,13,14]. In this model, which is one of several defining association of the enzyme to the outer mitochondrial membrane, the Type A sites are defined as mitochondrial sites from which the enzyme is released by the action of G6P, while the Type B sites are those at which HK-I remains bound even in the presence of G6P [11]. The latter has been shown to be affected by chaotropic salts such as KSCN [9]. HK _G6P _(A-HK-I) and HK_KSCN _(B-HK-I) represent HK-I released by treatment of mitochondria with G6P and by subsequent treatment with KSCN, and appear to be identical [9]. Results obtained on release of HK-I from these sites on mitochondria of various species suggested the possibility for the existence of distinct populations of the bound enzyme on the same mitochondria, differing in susceptibility to release by G6P. Furthermore, the two sites have been proposed to be located in membrane domains of different lipid composition, with the G6P-sensitive sites being in those enriched in acidic phospholipids [13]. Interaction of hexokinase with a number of subcellular (non-mitochondrial) structures have been found to take place in a G6P-independent manner [see [15] and references therein], further implying that binding of the enzyme to biological structures *may *occur in this manner.

In the present study, 2 mM G6P and 45 mM KSCN, were used to release HK-I from the two binding sites of rat, bovine and human brain mitochondria. The decision on the use of 45 mM KSCN which has established an important aspect of the present study, was based on the fact that the chaotropic salt, at the concentration utilized, did not cause any detectable disruption of the outer rat brain mitochondrial membrane, while, at the same time, caused measurable and reproducible release of HK-I. At concentrations higher than 55 mM, clear loss of mitochondrial integrity became obvious, based on activity determination of marker enzymes, as outlined in Methods. Also, KSCN at concentrations up to 60 mM, did not alter the binding capacity of rat liver mitochondria for HK-I (see Methods and Table 3). Regarding the effectiveness of a chaotropic salt such as KSCN in causing release of B-HK-I, it is intriguing that the partitioning of mitochondrially-bound hexokinase into hydrophobic (Triton ×-114) phase may be correlated with the proportion of the Type B sites [11]. This is apparently related to the type of the phospholipids present in membrane domains in which B-HK-I is located [11].

The most significant aspect of the present study is reflected by the fact that no rebinding of HK-I was detectable on mitochondria pre-treated with 45 mM KSCN (Table 2) in spite of measurable release of the enzyme (Table 1). Although use of a low concentration of the chaotropic salt served an important purpose in that it caused release while avoiding disruption of the mitochondrial membrane, the extent of release was not as high as one would have wished. Therefore, attempts were made toward enhancing release, while at the same time, keeping the integrity of the membrane structure. Amongst these strategies, use of a lower temperature for release by 45 mM KSCN (10°C instead of 30°C, based on the observations reported in reference [16]), and a mixture of KSCN and KCl (each at 22.5 mM concentration at 10°C), increased release from the usual 17% to 30% and 45%, respectively. Again, no binding could be detected. It is also noteworthy that use of MgCl_2_, at concentrations up to 6 mM resulted in no detectable rebinding, as observed in its absence (compare with the effectiveness of MgCl_2 _related to HK-I binding on site A, see, for example, [17]). Longer incubation times in the presence of KSCN did not result in greater release, suggesting that the KSCN-mediated process is at equilibrium.

The indication that site B is intrinsically incapable of rebinding was supported by repeating binding experiments with DCCD-blocked mitochondria following the procedure previously reported for mitochondria isolated from rat liver and hepatoma cells [6].

The fact that rebinding does not take place on site B would support the proposition that site A is the only physiologically-important site in relation to the release -rebinding of the enzyme shown to occur in response to the energy need of the brain [18]. This would not be surprising since G6P is probably the most important metabolite for HK-I in relation to structure-function of this allosteric protein in the brain.

In a study involving treatment of rat brain mitochondria with digitonin, two porin populations were suggested to exist in different membrane environments with regard to cholesterol [19]. One of these is thought to be located in a membrane environment relatively free of cholesterol (digitonin-resistant) and the other in a more cholesterol-rich domain (digitonin-solubilized). Furthermore, it has been suggested [19] that in rat brain mitochondria, hexokinase is predominantly located in relatively cholesterol-free domain. An increase in the level of cholesterol in biological membranes has been shown to coincide with fundamental changes including enhancement of rigidity and condensation [see for example [20] and references therein]. It may therefore be suggested that only in cholesterol-free domains, does the membrane have the required flexibility allowing for repeated release and rebinding of the enzyme at contact sites, controlled by the level of available G6P. Moreover, electrostatic interactions, an important component of this association [13], would probably be better facilitated at these sites.

If this is indeed the case, then what could be suggested as the reason for the presence of B-HK-I at such high proportions in bovine and human brain mitochondria [9]? Could it act as a reservoir providing "rate-limiting" A-HK-I, when in need? Accordingly, could it be suggested that A-HK-I being at limiting proportions in the human brain (about 20%) may exert a greater physiological role as compared to the situation (in rat brain mitochondria) where it is present in relative abundance (about 80%)? This is not inconceivable since redistribution of A-HK-I under certain experimental conditions has suggested a dynamic relationship between the Type A and the Type B sites [11], and the conformation of B-HK-I may alter upon removal of A-HK-I [14]. Furthermore, this specific observation is in line with our general understanding of the dynamics of biological membranes.

Based on the results presented here and the excellent earlier work of Wilson and co-workers [see for example [14] and references therein], it appears that the higher proportion of B-HK-I in the bovine and human brains, as compared to other mammalian sources, is not by coincidence. But, quite the contrary, it probably serves an important purpose in the overall context of the physiological role the association of hexokinase is thought to play in relation to regulation of glucose metabolism in the brain.

## Conclusion

In conclusion, the results presented demonstrate that the reversible association of type I hexokinase to the outer mitochondrial membrane is uniquely related to Type A site on the mitochondrion. Although a clear explanation for the role of HK bound to the type B sites cannot be offered with certainty, it can be suggested that the B sites may act as a reservoir of HK, furnishing the enzyme to the A sites when needed under high energy demands of the cell. More work would be required to substantiate this proposition.

## Methods

All biochemicals were obtained from Sigma Chemical Co. (St. Louis, MO, USA). Other chemicals were of reagent grade and products of Merck (Darmstadt, Germany). Rat brains were obtained from adult NMRI rats. Animals were killed by decapitation and the brains were rapidly removed and frozen immediately in liquid air where they were stored until use. Bovine brain was prepared from local slaughter house, and transferred to the lab in liquid air. Human normal brain tissue was obtained from neurosurgery department of a local hospital as biopsy specimen and transferred to the lab in liquid air, where they were stored until use.

Rat, bovine and human brain mitochondria were prepared as previously described [9]. Rat liver mitochondria were isolated according to Bustamante et al. [21]. Protein assay was done according to M-Katzenellenbogen et al. [22], and hexokinase activity was routinely determined as described previously [10]. Solubilization of mitochondrially-bound HK, determination of percentage release, and rebinding of the released (and re-concentrated) enzyme were carried out exactly as described by Kabir and Wilson [9]. The amount of solubilized activity is expressed as a percentage of the total activity in the mitochondrial fraction. In these experiments, the mitochondrial suspension was subjected to release with G6P (2 mM, pH 8.5) and after this step of solubilization, the resuspended pellet, in the same initial volume, was treated with 45 mM KSCN (pH 8.5, at 30°C, except otherwise indicated). The incubation time was the same as that used for G6P-induced solubilization, providing similar experimental conditions for the two releasing factors. In related experiments, this sequential process of release was reversed. Mitochondrial membrane integrity was confirmed by determination of the specific activities of marker enzymes: (malate dehydrogenase [23], glutamate dehydrogenase [24], rotenone- insensitive cytochrome c oxidase [25], citrate synthase [26] and adenylate kinase [27]) before and after treatment with KSCN (45 mM). Rat liver mitochondria were treated with KSCN at the chosen concentrations, and rebinding of rat brain HK-I on these preparations was performed as a control experiment.

Rebinding of HK was carried out after preliminary experiments for determination of appropriate ratios of HK: mitochondria. Data presented by Xie and Wilson were used as a guideline [28], thereby making sure that sufficient amounts of the enzyme was present for binding to any available sites on the mitochondria. To minimize the presence of any proteolytically-cleaved HK in these experiments, freshly-released (by G6P treatment) and concentrated enzyme was used. In brief, the G6P or KSCN-treated mitochondria (1 mg protein) were subjected to rebinding of released and concentrated enzyme (0.016 U) in total volume of 0.5 ml. Incubation carried out for 30 min on ice (in the presence of 2 mM MgCl_2_) was followed by centrifugation for 10 min using a microfuge. The pellet was resuspended in 0.5 ml of 0.25 M sucrose and assayed for bound hexokinase activity, or was subject to another release/rebinding regimen after washing (twice) with the same volume of 0.25 M sucrose. Reproducibility of the data presented in this manuscript was confirmed by repeating the experiments at least three times. Chemical modification by DCCD was performed by following a procedure described previously [6]. The observed pattern of dependency of loss of HK-I binding on the concentration of DCCD utilized, was found to be similar to those reported for mitochondria isolated from rat liver and hepatoma cells [6]. Routinely, a portion of these mitochondria was incubated for 15 min at room temperature with 0.01 mM DCCD (added from a 1 mM stock solution in methanol). Initial experiments (data not shown) indicated that DCCD at this concentration was maximally effective in blocking HK rebinding. The mitochondria were washed once with 0.25 M sucrose and used for the binding experiments. Control mitochondria were subjected to the same regimen except that pure methanol was used in place of the stock DCCD solution. DCCD did not affect hexokinase activity at the concentration utilized. Its effect on prevention of rebinding was performed according to Kabir and Wilson [9]. Briefly, mitochondria were subjected to HK release by G6P (2 mM). The pellet was resuspended in 0.25 M sucrose at 4 mg/ml (referred to as untreated).

## Competing interests

The author(s) declares that there are no competing interests.

## Authors' contributions

MN-G participated in the design of the study and finalized the manuscript. The work was carried out under his supervision. HR carried out most of the experimental work and participated in related discussions. AG conceived the study and participated in its design. He carried out the rebinding experiments and coordinated and helped to draft and finalize the manuscript. All authors read and approved the final manuscript.
